# Cardiovascular Mitochondrial Dysfunction Induced by Cocaine: Biomarkers and Possible Beneficial Effects of Modulators of Oxidative Stress

**DOI:** 10.1155/2017/3034245

**Published:** 2017-05-16

**Authors:** Manuela Graziani, Paolo Sarti, Marzia Arese, Maria Chiara Magnifico, Aldo Badiani, Luciano Saso

**Affiliations:** ^1^Department of Physiology and Pharmacology “Vittorio Erspamer”, Sapienza University of Rome, Rome, Italy; ^2^Drug Addiction and Clinical Pharmacology Unit, University Hospital Umberto I, Sapienza University of Rome, Rome, Italy; ^3^Department of Biochemical Sciences “Alessandro Rossi Fanelli”, Sapienza University of Rome, Rome, Italy; ^4^Sussex Addiction Research and Intervention Centre (SARIC), School of Psychology, University of Sussex, Brighton BN1 9RH, UK

## Abstract

Cocaine abuse has long been known to cause morbidity and mortality due to its cardiovascular toxic effects. The pathogenesis of the cardiovascular toxicity of cocaine use has been largely reviewed, and the most recent data indicate a fundamental role of oxidative stress in cocaine-induced cardiovascular toxicity, indicating that mitochondrial dysfunction is involved in the mechanisms of oxidative stress. The comprehension of the mechanisms involving mitochondrial dysfunction could help in selecting the most appropriate mitochondria injury biological marker, such as superoxide dismutase-2 activity and glutathionylated hemoglobin. The potential use of modulators of oxidative stress (mitoubiquinone, the short-chain quinone idebenone, and allopurinol) in the treatment of cocaine cardiotoxic effects is also suggested to promote further investigations on these potential mitochondria-targeted antioxidant strategies.

## 1. Introduction

Cocaine (COC) use has long been known to cause morbidity and mortality due to its cardiovascular toxic effects [1, 2]. COC can induce coronary and systemic vasoconstriction and arrhythmias, such as atrial and ventricular fibrillation [3], contraction band necrosis, atherosclerosis, and chest pain [4] as well as acute myocardial infarction [5], up to weeks after last consumption [6], even in presence of normal coronary arteries [7].

The pathogenesis of cardiovascular toxicity related to COC use has been reviewed recently [8–10]. Direct (block of voltage-dependent K^+^ and Na^++^ channels) and indirect (actions of catecholamines and their oxidation products on *α*- and *β*-adrenergic receptors) are suspected to be the primary pathogenic mechanisms.

The fundamental role of oxidative stress (OS) in COC-induced cardiovascular toxicity is well established [8, 11]. Moreover, formation and accumulation of reactive oxygen species (ROS) as a consequence of *α*- and *β*-adrenergic receptors stimulation [12, 13], as well as of enzymatic or nonenzymatic catabolism of catecholamines [14, 15], have been demonstrated in cardiac and vascular cells. Mitochondrial dysfunction leading to the production of ROS is implicated in cardiovascular toxicity [16, 17]. Furthermore, a number of drugs (e.g., anticancer drugs, antiviral drugs, oral antidiabetic drugs, and recreational drugs) have been demonstrated to induce toxic effects as a consequence of mitochondrial dysfunctions [18]. An important contribution of mitochondria in COC-induced OS and ROS production has been shown in experimental in vivo models [19, 20] and in the culture of rat cardiomyocytes [21]. In cardiomyocytes, the mitochondria themselves could become the target of COC-induced OS, due to ROS accumulation.

A better understanding of the role of mitochondrial dysfunction in COC-induced cardiovascular toxicity will help to select the most appropriate biological markers and to develop novel mitochondria-targeted antioxidant strategies. The purpose of the present paper is to review the state-of-the-art study of mitochondrial involvement in ROS production associated to COC-induced cardiovascular toxicity. In particular, we focused on the identification of possible biological markers of OS and the possible beneficial effects of OS modulators.

## 2. Mitochondrial Toxicity and Molecular Targets

The molecular mechanisms by which drugs of abuse, COC particularly, attack tissues' integrity is an issue of paramount importance. Pioneering experiments carried out using epithelial cell cultures [22] or animal models, such as mice [23, 24], have clearly suggested an involvement of mitochondrial chemistry based on the impairment of the respiratory chain with the rise of cytotoxic (ROS) species.

### 2.1. Mitochondrial Energy Production and Electron Transport Chain

In cells, most of the ATP is synthesized by the mitochondria via a proton electrochemical potential gradient, Δ*μ*H^+^ [25]. Under physiological conditions, the mitochondria are maintained operative by a resident mitochondrial DNA in synergy with nuclear DNA, both regulating fusion and fission and mitophagy dynamics of the organelles [26] and, indeed, the expression and activity of the respiratory chain electron transfer (eT) complexes. These complexes, at the level of the inner mitochondrial membrane, either collide among themselves randomly [27], the hypothesis later on reconsidered by [28], or are organised in supramolecular structures of the individual complexes [29]. Relevant to cell bioenergetics, the structural stability of the supercomplexes and the functional performance of the respiratory chain both have been shown to be modulated by the mitochondrial membrane potential [30] and the protein complexes phosphorylation. In addition and possibly related to the cocaine-dependent chemistry involving the proteo-membrane complexes, the functional performance of the supercomplexes has been shown to strongly depend on the membrane lipid composition and lipid peroxidation [31]. Regardless of whether organised as individual entities or as supercomplexes, the respiratory chain components enable the redox chemistry and the H^+^ translocation across the inner mitochondrial membrane to occur, ensuring the built up and maintenance of the proton-electrochemical gradient used by the mitochondrial ATPase to synthesize the ATP [25, 32].

Relevant to the COC-induced chemistry, the mitochondrial function appears affected both directly, particularly at the level of complex I [33], and indirectly, due to production of reactive oxygen and nitrogen species (ROS and RNS), both strongly affecting mitochondrial complex IV and permeability and fluidity of the membranes. The mitochondrial ATP synthesis depends on the cell metabolism, and the functional status of the molecular machinery is modulated at several levels, principally by the energetic demand and by the substrates availability; in this context, Ca^2+^ plays a crucial role.

### 2.2. Mitochondria and Ca^2+^ Homeostasis

In the frame of the molecular changes induced by COC, it is worth to recall its interference with the cell integrated Ca^2+^ signaling and homeostasis, whose network although intensively studied is still only partly understood [34]. A complex set of equilibria and chemical reactions tightly controls the flux of Ca^2+^ within all cell organelles and among specific molecular components of the extracellular and the intracellular cell compartments [34–37].

**Figure 1:**
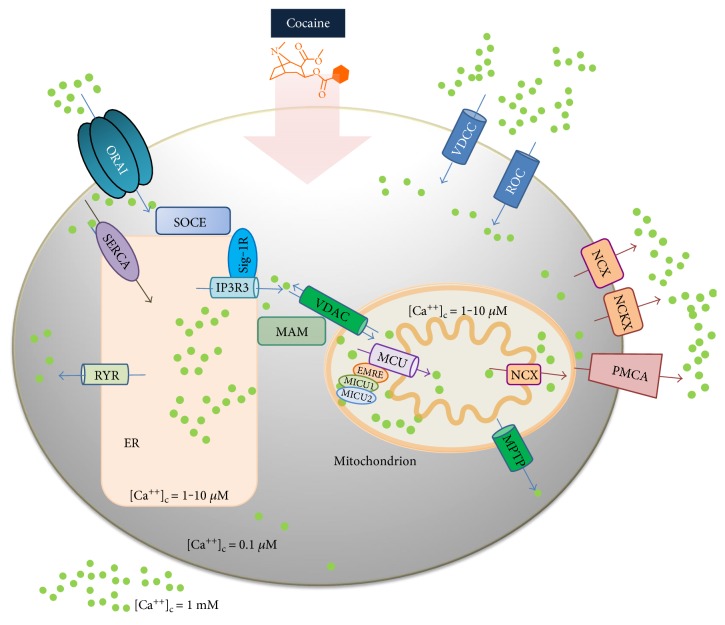
Main players of the cell Ca^2+^ molecular machinery as putative cocaine targets. Ideal intracellular Ca^2+^ concentration is maintained through complex equilibria among the extracellular space (1 mM), the cytoplasm (0.1 *μ*M), and the cellular stores (1.0–10 *μ*M), such as the mitochondrion, the endoplasmic reticulum (ER), the Golgi apparatus, and nucleus. The ion trafficking occurs via a variety of selective membrane channels, Ca^2+^-binding proteins and transporters and ion exchangers and receptors, altogether responsible for Ca^2+^ import, export, and homeostasis. Import occurs at the level of (i) cell plasma membrane through the calcium release-activated Ca^2+^ channel protein 1 (ORAI1), the store-operated calcium entry channels (SOCE), and specific receptor-operated channels (ROC) such as AMPA, NMDA, TRPC, and the voltage-dependent calcium channels (VDCC); (ii) endoplasmic reticulum (ER) through the sarco/endoplasmic reticulum calcium ATPase (SERCA); (iii) mitochondria intermembrane space through the voltage-dependent anion channel (VDAC); and (v) in the matrix by the mitochondrial uniporter (MCU), in synergy with the mitochondrial calcium uptake (MICU) system. Extrusion occurs at the level of (i) cell plasma membrane mainly by the plasma membrane calcium ATPase (PMCA) and the sodium calcium exchangers (NCX) also potassium-dependent (NCKX) and (ii) the ER by the ryanodine (RYR) and the inositol 1,4,5-trisphosphate receptors (I P3R), as well as by the mitochondrial permeability transition pore (MPTP).

In the extracellular space, Ca^2+^ concentration is in the mM range, whereas in the cytoplasm of a resting cell is ~10^2^ nM [38]. The large concentration gradient is maintained by a dynamic equilibrium, involving a finely integrated Ca^2+^ controlling molecular machinery [34, 39] including a variety of plasma membrane Ca^2+^ channels, receptors, exchangers, pumps, binding proteins, chaperons, and transporters (Figure 1).

In the cell, the intracellular Ca^2+^ ions are accumulated into specific cell compartments, the so-called Ca^2+^ stores. These are the endoplasmic/sarcoplasmic reticulum (ER, for simplicity), the mitochondria, and to some extent the Golgi apparatus, together with the cell nucleus and other organelles, such as the lysosomes and peroxisomes. The Ca^2+^ concentration in the stores may rise up to 1 or 2 orders of magnitude (1–10 *μ*M) higher than that in the cytoplasm, the value depending on the actual cell compartment, and its functional state [40–42].

The mitochondria and ER are responsible for the accumulation in the stores of most Ca^2+^ contributing, respectively, to microcompartmentalization of up to 25% and 75% Ca^2+^. These compartments are tightly interconnected at specialised sites named *mitochondrial-associated membranes* (MAM). At this level, the side-by-side proximity between ER and the mitochondria allows the transfer of Ca^2+^ ions to the mitochondria from proteins and chaperons of the ER. This process occurs via specific channels such as the inositol-tri-phosphate receptors and the ryanodine receptors. At the level of the outer mitochondrial membrane (OMM), Ca^2+^ ions are transported from the cytoplasm into the intermembrane space (IMS) through the voltage-dependent anion channel (VDAC): this reaction uses ATP; thus, once promoted, ATP synthesis is stimulated. The transport, across the inner membrane of Ca^++^ from the IMS to the mitochondrial matrix, is mainly contributed by the mitochondrial calcium uniporter (MCU), an ion channel that selectively drives the Ca^2+^ entry into the matrix. Transport occurs in synergy with two complexes named the mitochondrial Ca^2+^ uptake 1 (MICU1) and the mitochondrial Ca^2+^ uptake 2 (MICU2), together setting the threshold for the Ca^2+^ uniporter activity, also mediated by the essential MCU regulator (EMRE) [43]. It is worthy to point out that the mitochondrial Ca^2+^ loading and its back release to ER take part in the physiological, vital ion-buffering system, while the mitochondrial Ca^2+^ overloading most often paves the way to apoptosis or even to cell irreversible damage.

Uncontrolled mitochondrial Ca^2+^ accumulation rapidly induces, in fact, a decrease of the mitochondrial membrane potential (Δ*Ψ*) leading to pathological production of ROS, RNS with opening of the mitochondrial permeability transition pore (MPTP), and release of cytochrome *c* and other proapoptotic components. The Ca^2+^ extrusion into the extracellular compartment, therefore, is also under tight control: it occurs via (i) the plasma membrane-associated Ca^2+^ ATPase pump (PMCA), extruding against an unfavourable electrochemical gradient, 1 Ca^2+^ ion per hydrolysed ATP and (ii) the potassium-independent Na^+^/Ca^2++^ exchanger (NCX) and the potassium-dependent exchanger (NCKX) [44].

According to recent reports [45, 46], COC interferes with the intracellular Ca^2+^ distribution and trafficking. The interference has been proposed to occur at the level of the store-operated calcium entry (SOCE) system and particularly at the sigma-1 receptor site (Sig-1R) [47]. This is an intracellular chaperone embedded in the endoplasmic reticulum and is responsible for Ca^2+^ loading into the intracellular stores, the mitochondria included. The functional activity of Sig-1R was shown to be depressed by COC with impairment of the Ca^2+^ equilibrium among the cell cytoplasm and stores [40, 48]. It is worth noticing that the inhibition of SOCE via COC binding at the Sig-1R [45], if confirmed, might lead to even opposite pathophysiological effects. Depending on the extent of binding and duration of SOCE inhibition, the electrophoretic transfer of the positively charged Ca^2+^ in the mitochondrial matrix could, initially, lead to a slight depression of the mitochondrial Δ*Ψ*, counterbalanced by stimulation of ATP synthesis. No wonder, therefore, the COC induced mitochondrial Ca^2+^ loading might be overlooked. On the opposite, the persistence of the mitochondrial Ca^2+^ loading leads to the opening of the MPTP and triggers the apoptotic programme, with release of cytochrome *c* and other proapoptotic components [34, 37, 49–52]. Accordingly, COC-treated rat astroglioma cells had shown a dose-dependent manner depression of mitochondrial Δ*Ψ* and a disruption of cell morphology [53].

### 2.3. Mitochondria, ROS, and RNS Production in the Pathogenesis of Cardiovascular Toxicity

Cardiomyocytes undergo incessant contractions, their mitochondria requiring a regular supply of O_2_ and reducing substrates. Normally, during mitochondrial respiration, the vast majority of O_2_ is reduced to water via the electron transfer (eT) chain (4e^−^/O atom), and only a small oxygen amount (0.1–2%) undergoes a 1- or 2-electron reduction, with formation of highly reactive partially reduced species, among which H_2_O_2_ and the superoxide radical ion (O_2_^−·^) are the best representatives. These, when produced at sub-*μ*molar, nanomolar levels contribute to the formation of the cellular pool of physiological ROS that plays crucial signaling roles in a variety of conditions. Similarly, under normal conditions, also a number of more or less stable nitrogen oxides can be detected in the cells and tissues (RNS). These include nitric oxide (NO) and peroxynitrite (ONOO^−^), that is, highly reactive species responsible, particularly the latter, for cell redox reactions that are often highly detrimental, such as protein nitrosation and membrane nitration. Among them, NO is present in the environment at up to nanomolar concentrations, as produced by the cell constitutive NOSs (eNOS and nNOS).

When present in large excess by the inducible iNOS (≥*μ*M), NO is a potent inhibitor of the mitochondrial respiration [54]. Noticeably, in the presence of enough O_2_ (5 ÷ 20 *μ*M) and a suitable electron flux through the respiratory chain sustained by the mitochondrial substrates and reduced cytochrome *c*, the presence of nanomolar NO does not depress (significantly) cell respiration. Interestingly, from the bioenergetics signaling point of view, under these conditions, the apparent affinity for O_2_ (*K*_*M*, O2_) of cytochrome *c* oxidase (CcOX) rises [55], and the mitochondria become sensitive to the O_2_ concentration, thus ready to shift to glycolytic production of ATP [56, 57]. Under persistent hypoxic conditions, when the mitochondrial respiratory chain experiences for longer times a too low (insufficient) O_2_ concentration, a different landscape could be depicted. The rapid activation of constitutive NOS is observed together with the rise of NO concentration, whose increase, however, induces a depression to the oxidative phosphorylation due to, particularly, the inhibition of not only complex IV but also to some extent of complex I [57]. As observed in neurons, glycolysis takes place to compensate for the decreased ATP synthesis, a finding not directly shown, however, in cardiomyocytes. In addition, the cell environment turns acidic facilitating the conversion of nitrite (NO_2_^−^) into nitric oxide. NO, in the presence of O_2_^−^, at a diffusion-limited rate [58], forms the highly cell detrimental peroxynitrite, ONOO^−^, initiating and sustaining a vicious circle that leads to permanent blockage of the mitochondrial eT [59, 60]. At this point, the reaction mechanisms controlling the cell steady-state level of ROS and RNS might become severely insufficient.

In summary, due to complex I inhibition by COC, the cardiomyocytes are likely called to face in rapid sequence, though not necessarily in this chronological order, hypoxia and cell acidification and rise of ROS/RNS species. As a consequence, cell survival might be at risk and cell death committed. Most frequently, the chemical species formed are strong oxidizing agents such as hydrogen peroxide, hypochlorous acid and peroxynitrite ion, and some of them are radical, for example, the nitric oxide and superoxide anion or the hydroxyl radical. Pathophysiologically relevant, not only cardiomyocytes but also the endothelial cells and the leucocytes, activated during the oxidative burst and the inflammation response, are responsible for the environmental physicochemical change and the uncontrolled ROS and RNS production. Altogether, the events point to excess ROS/RNS as being responsible for the production of cell detrimental effects, thus linking together, at the mitochondrial level, the OS, the early inflammation response, and cell death.

### 2.4. Crosstalk between the NO Chemistry and Cocaine

**Figure 2:**
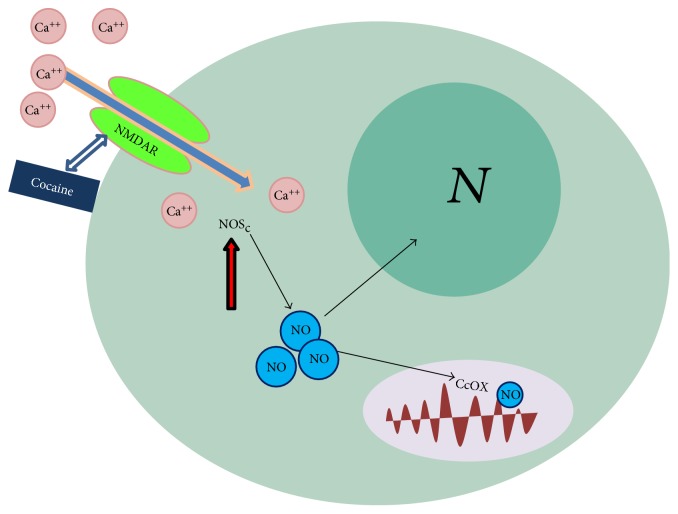
NMDA-receptor targeting by cocaine (hypothesis). The scheme is drawn by analogy to the functional effects observed at the level of the cell nitric oxide chemistry and detected when treating glioma cells in culture with morphine [55]. It shows the activation of the cocaine-mediated NMDA-R, leading to cytoplasmic Ca^++^ rise, activation of the constitutive NOS, and release of NO, targeting mitochondrial respiratory chain complexes.

NO is actively produced by the NOSs [61] or it is chemically generated all throughout our organism. Three NOS isoforms have been identified and named after the cell tissues where they were first detected: the endothelial NOS (eNOS) from the endothelium, the neuronal NOS (nNOS) from the nervous system, and the inducible NOS (iNOS) from immunocompetent cells. These three isoforms share a substantial sequence homology (50–60%) and some basic features such as one catalytic Fe metal, the cofactors, and the substrates' binding sites. The expression and activity of the iNOS strongly depend on cell stressors but are independent on cell pCa^2+^ whereas eNOS and nNOS, both constitutive enzymes, are finely regulated by the concentration of cytosolic Ca^2+^. Relevant to the NO chemistry, COC is known to react also with the NMDA receptor [62], whose activation is induced via Ca^2+^ rise, activity of nNOS, and NO production [63] (Figure 2()); a pathway was also reported for morphine [55].

NOSs use arginine and O_2_ as substrates. The affinity for O_2_ is not equally distributed among the NOS isoforms. The eNOS shows the highest affinity (*K_M_*~5 *μ*M), comparable to that of the mitochondrial CcOX, while nNOS and iNOS have a lower O_2_ affinity [57, 64].

Under hypoxic conditions, therefore, the O_2_ availability can limit the enzymatic production of NO by the NOSs. During hypoxia, maintenance of the NO homoeostasis may require the release of NO from bulk nitrosothiols, or at the expenses of metal ions (Fe^2+^ and Cu^+^) bound to proteins or free in solution. These ions catalyze the reduction of nitrite to NO and particularly at acidic pH and under hypoxic conditions [65].

In agreement with previous reports [66–71], the involvement of the NO chemistry in the development of COC addiction has been recently confirmed by the results of the selective 7-nitroindazole (7-N) inhibition of nNOS, induced on Wistar rats. The animals when preliminarily treated with 7-N showed a significant attenuation of the COC withdrawal symptoms, and their brain-isolated synaptosomes displayed both the reversal of the drug mitochondrial depression and the decrease of GSH levels [72]. The fundamental role of mitochondrial GSH in protecting membrane functions was also observed in an experimental model of COC-induced hepatotoxicity in rats [73].

In humans, PET measurements performed using C-11-COC have shown in the early 90s that COC redistributes in most organs and tissues although following different kinetics (from seconds to several minutes) [74]. Redistribution likely includes the skin, and heavy COC abusers often display unpleasant skin signs, whose molecular mechanisms, however, are still mostly obscure. In this framework, it may be worthy to recall that the systemic administration of COC to male Sabra rats, thereafter subjected to skin biopsies, was able to rise the iNOS and xanthine oxidase (XO) activity prevented by specific inhibitors, such as the L-nitroso-arginine methyl-ester (L-NAME) and the oxypurinol (OP), respectively; the same authors reported similar results using human keratinocytes in culture [75]. The proposition put forward was that the oxidative-oxynitrosative damage was bound to the skin accumulation of superoxide and nitric oxide radicals, readily forming peroxynitrite [58] and lipoperoxides, along with a marked decrease of ROS/RNS scavengers such as reduced glutathione (GSH) and ascorbic acid (AA) [75]. This hypothesis appears fully consistent with the suggestion that the COC oxidative metabolites, and among them particularly, the nitrogen N-derivatives, are involved in the adverse biological effects observed in the human body, at least when chronically exposed to COC [76].

## 3. Mitochondria and Cocaine-Induced Cardiovascular Toxicity

**Figure 3:**
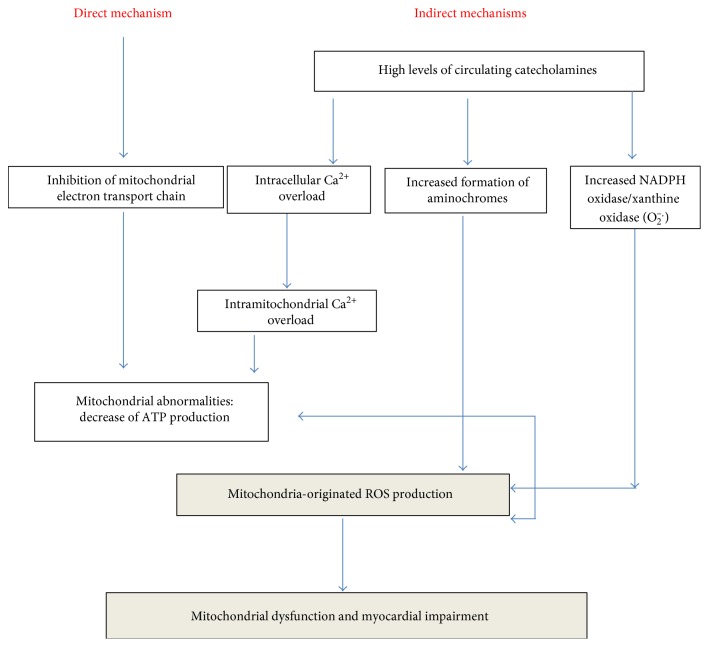
Cocaine-induced mitochondrial dysfunction.

As mentioned above, the role of mitochondria in the pathogenesis of COC-induced cardiovascular toxicity is well recognized [8, 20, 21, 33, 77]. COC may induce mitochondrial dysfunction in cardiomyocytes and in endothelial cells, based on direct and indirect mechanisms (Figure 3). Owing to its pathophysiological relevance at both cardiomyocytes and endothelial level, it may be worth to summarize the evidence supporting the hypothesis that COC is likely responsible for a specific mitochondrial impairment.

### 3.1. Cardiomyocytes

Notably, stimulation (and overstimulation) of *β*-adrenergic receptors (*β*-AR) triggers the release of Ca^2+^ in the mitochondria [78]. Indeed, stimulation of *β*-adrenergic receptors increases Ca^2+^ levels in the cytosol, through the activation of protein kinase A (PKA): increased cytosolic Ca^2+^ leads in turn to phosphorylation of Ca^2+^-protein substrates and to the transfer of Ca^2+^ into the mitochondria [79]. As mentioned above (Section 2.2), excess mitochondrial Ca^2+^ impairs ATP production, causing nitro-oxidative stress with changes in permeability of the mitochondrial membrane, altogether leading to structural degeneration of cardiomyocytes [9, 80, 81]. Overproduction of mitochondrial ROS/RNS is in fact responsible for the massive opening of MPTP [16, 82] resulting in a further dysfunctional and structural degeneration of these organelles.

As already mentioned in Section 2.1 in isolated brain and liver mitochondria [83] and in culture rat myocardial cells, COC at high concentration had shown to inhibit complex I (NADH dehydrogenase) activity [33] leading in turn to inhibition of ATP synthesis. Accordingly, Fantel and colleagues [84] demonstrated a COC inhibitory effect on mitochondria respiration in rat embryo tissues. Importantly, in a model of myocardial ischemia-reperfusion, it has been demonstrated that a reduction in complex I activity may enhance ROS production by complex III [85].

A further mitochondrial role in COC myocyte toxicity is suggested by experimental studies, in which a mitochondria-dependent apoptosis was observed [77, 86, 87]. Indeed, in chronic COC-treated rats [77] and in cultured fetal [87] and adult [86] myocytes, COC induced apoptosis. The cytotoxic effects on cardiomyocytes were related to the release of cytochrome *c* from the mitochondria with activation of caspase-9 and caspase-3, whose inhibition blocked cell apoptosis [87]. Accordingly, a COC-induced apoptosis associated with the release of cytochrome *c* was observed also in cultured bovine coronary artery endothelial cells [88]. Interestingly, from the mechanistic point of view, in experiments carried out using adult rat ventricular cardiac myocytes, the caspase inhibition decreased the *β*-AR-stimulated apoptosis [86].

Apoptosis activation was also observed in the cerebral cortex of human COC addicts [89]. The postmortem brain study showed a significant reduction in the content of mitochondrial cytochrome *c* in prefrontal cortex: the authors suggest that the downregulation of cytochrome *c* could represent the induction of a counter regulatory adaptation to brain apoptotic effects induced by COC via mitochondria oxidative stress.

As mentioned above (Section 2.3), the accumulation of ROS/RNS is an important event by which COC may induce mitochondrial dysfunction with subsequent cardiotoxicity. Although the extent of mitochondrial dysfunction produced by COC is still unknown, it is generally accepted that the mitochondria are the main source of ROS production [90, 91] meantime being targets of the oxidative stress.

COC-induced ROS production may occur by mechanisms different from electron leak at the sites of the respiratory chain complexes, namely, by

- formation of O_2_^−·^, during catecolamine oxidation (intramitochondria redox cycling),
- synthesis of H_2_O_2_ by monoamine oxidase (MAO), during oxidative deamination of catecholamines (outer membrane of mitochondria),
- ROS-induced ROS mitochondria formation [92].

Thus, ROS formation has been associated with COC-induced catecholamine release [8, 93].

As noted, a crucial role in COC-induced toxicity is played by transformation of catecholamines into aminochromes, that is, the oxidative catecholamine metabolites [93]. Indeed, when the level of catecholamines rises and the enzymes responsible for their catabolism become less efficient, as it might likely occur during COC abuse, catecholamines can undergo oxidation [14, 94] with formation of aminochromes (adrenochrome, dopachrome, and noradrenochrome); these molecules are very active from the redox cycling point of view. In bovine heart, it has been demonstrated that adrenochrome is reduced into semiquinone by mitochondrial complex I [95] inducing in cardiomyocyte mitochondria the formation of O_2_^−·^ [9, 94, 96].

Genova and coworkers [97] had shown that mitocondrial complex I is involved both in initial generation of superoxide and in the reduction of adrenochrome to its semiquinone form. Furthermore, the superoxide anion O_2_^−·^ in turn increases the adrenaline oxidation rate [14, 97]. Thus, the mitochondria, on one side, are largely responsible for cardiomyocyte oxidative stress, while on the other side, they are themselves targets of the stress. In addition, it is worth mentioning that the adrenochrome inhibits the oxidative phosphorylation of cardiac mitochondria [98] and leads to further enhancement of mitochondrial impairment.

Also MAO, flavin enzymes located in the outer membrane of mitochondria, are responsible for oxidative deamination of catecholamines, resulting in synthesis of H_2_O_2_ leading in turn to highly reactive HO· [93, 99]. Accordingly, in an experimental model in rats, it has been demonstrated that myocardial oxidative stress could be partially prevented by MAO inhibitors [100].

A further contribution to the stimulation of COC-induced mitochondrial ROS production may be derived from NADPH oxidase (Nox) and XO activity, also contributing to ROS generation in cardiac tissue [8, 13]. Indeed, the *α*1-adrenoceptor stimulation increases the activation of Nox [13, 101] which in turn produces O_2_^−·^.

In an in vivo model of COC-induced diastolic dysfunction, it has been shown that 7 days of COC administration induces an increase of mitochondrial ROS production in cardiac fibers, with uncoupling of mitochondrial respiration [20]. It is worth noticing that over a similar period of incubation COC administration induces also the activation of Nox and XO, whose functional onset might precede mitochondrial failure [102]; this finding suggests that it is the ROS production by the Nox and XO that first triggers the ROS production by mitochondria not vice-versa [19].

Accordingly with this hypothesis, it has been suggested that MitoQ [20] and allopurinol [19] treatments may prevent oxidative stress and attenuate COC-induced cardiotoxicity.

### 3.2. Endothelial Cells

In endothelial cells, the mitochondrial content is reduced, compared to other cell lines [103, 104]. Thus, by comparison with cardiac myocytes and other cell types characterized by higher energy requirements, one might expect, from the quantitative point of view (only), a relatively smaller production by mitochondria of COC-induced reactive species. This notwithstanding ROS production by endothelial cell and their contribution to development of heart disease [104] has been demonstrated in rat [105] and in mouse [106] models. In cultured endothelial cells used as experimental models of ischemia/reperfusion, extensive amounts of ROS were observed [107].

The pro-oxidant activity of XO has been observed [108], and XO from endothelial vasculature has been proposed as the main ROS enzymatic source [93]; accordingly, patients with ischemic cardiomyopathy oxypurinol-induced inhibition of XO had shown improved myocardial contractility [109]. In endothelial cells, the activity of XO increases in I/R and it is a source of O_2_ when in the presence of high levels of hypoxanthine.

## 4. Possible Biomarkers of Cocaine-Induced Oxidative Stress

Notably, biological markers (biomarkers) may be useful to quantify biological processes, disease state, or therapy prediction and therapeutic tools [110, 111]. The increase in understanding mechanisms of oxidative stress in drug [8, 112, 113] and alcohol [114, 115] addiction has led to identify oxidative stress markers, that, although not validated and specific, could help to evaluate oxidative status in drug abusers, both in acute and chronic use and in withdrawal syndrome [116, 117]. In Table 1, the proposed peripheral biomarkers of OS and relative references are listed.

In a recent study [117], it has been suggested that thiobarbituric acid reactive substances (TBARS) and brain-derived neurotrophic factor (BDNF) could be biomarkers for evaluation of severity of crack COC use. Furthermore, the authors found in male crack COC users a positive correlation between TBARS levels and severity of abuse during withdrawal syndrome. Notably, TBARS are an aspecific biomarker of peripheral oxidative stress, consisting of a quantification method for malondialdehyde (MDA) and stabile product of lipid peroxidation [118, 119]. Accordingly with clinical data, experimental studies in rats showed an increase in MDA levels in the heart, both after COC self-administration and extinction training [120] and after COC injection [121, 122].

Conversely, in a clinical study aimed at evaluating total antioxidant capacity in COC and methamphetamine subjects [123], no difference was found in MDA blood levels with respect to control. One possible explanation is that discrepancy in results may be due to differences in some characteristics of participants. Indeed, Sordi and coworkers [117] included subjects (*N* = 49) positive for current COC use, while in the study from Walker and coworkers [123], patients (*N* = 126) had used COC within 60 days prior to the test and almost 23% were positive for use. It can be suggested that the oxidative damage of lipids produced by current COC use, counteracting by antioxidant defense (see below), may shift towards antioxidant systems when the subjects became progressively abstinent.

Even though TBARS are neither specific nor quantitative [119], given that MDA plasma levels showed an increase in acute myocardial infarction [124, 125] and also in brain illnesses such as Parkinson's [126] and Alzheimer's [127] diseases, this biomarker may be useful to assess relative level of lipid peroxidation in COC abusers.

An increase in BDNF levels among crack users with respect to control subjects was also found [117, 128]. The function of this peripheral brain injury biomarker in drug-induced neuroadaptation is well known [129], and recent clinical data in chronic schizophrenia patients [130] showed that both BDNF and OS may be involved in the pathophysiology of this disease, suggesting an interaction mechanism between oxidative damage and neurotrophin dysfunction. Further investigations implicating these two peripherally measured biomarkers should contribute to understand the relative implication and interaction of oxidative stress and neurotrophic factors in disorders.

## 5. Antioxidant Defense System Biomarkers of Cocaine Oxidative Stress

Blood peripheral biomarkers of antioxidant enzymes were evaluated in a population of COC user [123], (*N* = 126; 18% abstinent for 1 month and more prior to the inclusion). The results showed no differences in the activities of glutathione peroxidase and catalase between COC user and control subjects, whereas a significant reduction in the SOD activity was observed in erythrocytes.

Accordingly, in a rat model of COC-induced heart injury, Moritz and coworkers [122] had shown that COC long-term administration caused a significant decrease in SOD activity; a biphasic trend in SOD concentration in rat spleen was observed after chronic COC administration in vivo [131] since that, after an early peak, SOD was significantly depleted 24 hours after COC treatment.

Notably SOD are an ubiquitous family of enzymes [132, 133] in which actually three distinct isoforms has been identified in humans. SOD1 (Cu/ZnSOD) is the major intracellular form of SOD, accounting for almost 80% of total SOD protein and is localized to the outer mitochondrial membrane, while SOD2 (MnSOD) is localized exclusively in the mitochondrial matrix [134, 135] and is expressed in the heart, lung, liver, and blood cells. SOD3 is the major SOD of human extracellular matrix of different tissues, mainly expressed in the lung and scarcely in the brain [136].

In the rat liver, SOD1/Cu, Zn was found in the mitochondrial intermembrane space and SOD2 was found in the matrix and also in the inner membrane [137]. Recent experimental data in rats [120] indicate no changes in SOD activity (irrespective of the isozyme subclass) in peripheral organs such as the heart and the liver, both during COC self-administration and during extinction phase (10 days). Conversely, a significant enhancement in SOD activity was found in the hippocampus and in the kidney. The authors suggest that different changes in the activity of SOD in rat brain structures and peripheral tissues may reflect differences in OS status and that increases in the SOD enzymatic activity could correspond to a reduction in MDA concentrations.

Due to its exclusive nuclear-encoded localization in the mitochondrial matrix, SOD2 is considered the main mitochondria antioxidant defense against toxic effects of ROS. So it can be suggested that evaluation of activity of SOD with respect to its isozyme subclasses could be a more specific biomarkers of mitochondrial oxidative stress. A further attention to different isozyme overexpression in specific cell types and tissues may achieve a contribution to better identify specific targets of oxidative stress.

Another peripheral biological marker that might reflect oxidative status in tissues is the level of glutathionylated hemoglobin (Hb). The role of the mechanism of S-glutathionylation (i.e., the conjugation of glutathione to protein cysteine residues catalyzed by glutathione S-transferase P) in response to oxidative stress in drug addiction was discussed in a recent review [113]. Protein S-glutathionylation can be considered a protective mechanism associated with elevated oxidative stress in alcohol, heroin, and also in COC abuse. In preclinical studies, acute and chronic COC treatment, but not withdrawal, had shown to increase brain formation of glutathionylated protein and a decrease in expression of GSH-S-transferase P [138, 139]. Notably most of the glutathionylated proteins are intracellular [110]; to date, in human, the extent of glutathionylation in some pathologies (i.e., diabetes mellitus, hyperlipidemia, and uremia) can be measured only in blood [140], since in red blood cells Hb accounts for 97% of protein composition [141]. Importantly, increased levels of glutathionylated Hb were observed in cigarette smokers [142, 143] suggesting that its quantification can be used as a low-invasive clinical biomarker of oxidative stress-associated diseases. To our best of knowledge, no clinical data are present in literature regarding glutathionylated Hb in COC addicts.

In conclusion, we further retain that in studies concerning the evaluation of oxidative stress in drug abuse and clinical relevance of relative biomarkers it is important to take into account factors that could significantly influence both oxidative state and antioxidant defense. Physiological (e.g., age, gender, body weight, diet, and lifestyle) and pathologic factors (psychiatric comorbidity, cardiovascular and metabolic illnesses and their relative severity, etc.) as well as drug abuse history [age of onset, duration, polydrug abuse, tobacco smoking, alcohol use, and prevalence of current use (i.e., during last month)] could affect total oxidative state. Only early patient stratification based on their profile could help to identify the most appropriate panels of both diagnostic and prognostic biomarkers and conduct to optimal management for the patients.

## 6. Modulators of Oxidative Stress in Mitochondrial Protection: Future Direction

The use of antioxidants as therapeutic tools is still controversial [8, 144, 145]. In a recent review on potential use of modulators of OS in treatment of COC cardiotoxic effects, Graziani and coworkers [8] underlies that both preclinical and clinical data in literature has not yet been adduced to argue conclusive evidence.

However, the fundamental role of mitochondria in COC-induced OS strongly suggests that mitochondria-targeted intervention could become a pharmacological strategy to prevent and to treat this kind of damage. In Table 2, selective antioxidant compounds for potential therapeutic use in COC toxicity are reported.

In an experimental model of COC-induced cardiac dysfunction, *MitoQ* (mitoubiquinone) had shown to limit COC-induced left ventricular dysfunction [20]. Accordingly, in vitro studies [146, 147] of rat-stretched cardiomyocytes showed that MitoQ could prevent both mitochondrial damage and increase in XO activity and protect mitochondrial membrane potential. MitoQ is actually the most studied mitochondria-targeted antioxidant therapeutic compound [148] and some human studies have confirmed its efficacy in some cardiovascular pathologies, such as hypertension, and drug toxicity (alcohol, adriamycin) [47]. To our best of knowledge, no human studies are present in literature on the potential effects of MitoQ in treatment of COC-induced mitochondria toxicity.

On the basis of the abovementioned mechanisms of mitochondria dysfunction induced by COC, other therapeutic tools may be hypothesized.

The short-chain quinone *idebenone* has been also suggested to be beneficial in mitochondrial dysfunction [149] due to its antioxidant effects [150]. Idebenone proved to rescue ATP levels under conditions of impaired complex I transferring electron in mitochondrial respiratory chain from complex III [151]. The COC toxic effect of inhibition of the activity of mitochondrial complex I [33] may be reversed by some short-chain quinones. Consistent with this capacity of ATP activity rescue, idebenone should be investigated as a possible treatment for COC-induced dysfunction in mitochondrial respiratory chain [152].

In an experimental model of COC-induced diastolic dysfunction, XO activity inhibition by *allopurinol* [19] had preserved both left ventricular systolic and the decrease in ATP production, confirming the contribution of COC-induced mitochondrial ROS production in cardiac tissue. The protective role of allopurinol was confirmed in human and rat left ventricular (LV) myocytes with volume overload where the increase in ATP demand and the concurrent XO-mediated ROS can decrease mitochondrial respiration and contractile function [153] and in remodeling processes after experimental myocardial infarction [154]. To date, some clinical data appear to suggest that pharmacological XO inhibition could represent potential tools for the treatment of human cardiomyopathy [155]. The safety profile of (the old) allopurinol underlies the possibility of testing this XO inhibitor for further therapeutic interventions.

## 7. Conclusion

In the present paper, the role of cardiovascular mitochondria in COC-induced OS and ROS production was reported. Preclinical and clinical data underlie the fundamental participation of mitochondrial dysfunction to pathogenesis of COC-induced cardiovascular toxicity. As a consequence, possible biological peripheral markers of OS mitochondrial injury may be proposed. Both the antioxidant defense system biomarkers SOD2/MnSOD and glutathionylated Hb appear to be appropriate peripheral biomarkers of oxidative stress: since clinical data in COC and psychotropic drug users are inadequate to draw any conclusion, it could suggest that additional studies in this population subjects may be performed. Even in the case of potential therapeutic effects of mitochondrial protection, further studies on the proposed antioxidant drugs (MitoQ, idebenone, and allopurinol) will be crucial to assess their effectiveness or inability to counteract mitochondrial dysfunctions induced by cocaine.

## Figures and Tables

**Table 1:** Peripheral biomarkers of cocaine-induced oxidative stress.

Markers	Sample	Note	References
MDA	Plasma	Aspecific biomarker of lipid peroxidation	[117, 120–123]
TBARS	Plasma	Aspecific biomarker of lipid peroxidation	[117, 128]
BDNF	Plasma	Negative correlation with severity of cocaine use	[117, 128]
Glutathionylated Hb	Plasma (RBC)	Increased levels in oxidative stress conditions (i.e., cigarette smokers)	[113, 140]
SOD	Plasma (RBC)	Decreased levels of activity	[120–122, 131]

BDNF: malodialdehyde; TBARS: thiobarbituric acid reactive substances; BDNF: brain-derived neurotrophic factor; Hb: hemoglobin; RBC: red blood cells; SOD: superoxide dismutase.

**Table 2:** Potential therapeutic use of selective antioxidant compounds for cocaine-induced mitochondrial impairment.

Antioxidant compounds	Mechanism of action	References
MitoQ	Inhibition of XO activity and protection of mitochondria membrane potential	[20, 47, 148]
Idebenone (short chain quinone)	Transferring of electron in mitochondrial respiratory chain from cytoplasm to complex III (bypass deficiency in complex I)	[149–152]
Allopurinol	Inhibition of XO activity and consequent rescue of in ATP production	[19, 153–155]

## Abbreviations

AA: Ascorbic acid
*β*-AR: *β*-Adrenergic receptors
BDNF: Brain-derived neurotrophic factor
CcOX: Cytochrome c oxidase
COC: Cocaine
Δ*Ψ*: Mitochondrial membrane potential
eNOS: Endothelial NOS
ER: Endoplasmic/sarcoplasmic reticulum
eT: Electron transfer
GSH: Glutathione
Hb: Hemoglobin
H_2_O_2_: Hydrogen peroxide
HO·: Hydroxyl radical
HO: Heme oxygenase
iNOS: Inducible NOS
IMS: Intermembrane space
L-NAME: L-Nitroso-arginine methyl-ester
MAM: Mitochondrial-associated membranes
MAO: Monoamine oxidase
MDA: Malondialdehyde
MCU: Mitochondrial calcium uniporter
MICU1: Mitochondrial Ca^2+^ uptake 1
MICU2: Mitochondrial Ca^2+^ uptake 2
MitoQ: Mitoubiquinone
MPTP: Mitochondrial permeability transition pore
NAC: N-Acetylcysteine
nNOS: Neuronal NOS
NO·: Nitric oxide
Nox: NADPH oxidase
NMDA: N-Methyl-D-aspartate
NOS: NO synthase
O_2_^−·^: Superoxide anion
OMM: Outer mitochondrial membrane
ONOO-: Peroxynitrite ion
OP: Oxypurinol
OS: Oxidative stress
OXPHOS: Oxidative phosphorylation
PKA: Protein kinase A
PMCA: Plasma membrane associated Ca^2+^ ATPase pump
RNS: Reactive nitrogen species
ROS: Reactive oxygen species
Sig-1R: Sigma-1 receptor site
SOCE: Store-operated calcium entry
SOD: Superoxide dismutase
TBARS: Thiobarbituric acid reactive substances
XO: Xanthine oxidase
EMRE: Essential MCU regulator
VDAC: Voltage-dependent anion channel
NCX: Potassium-independent Na^+^/Ca^2++^ exchanger
NCKX: Potassium-dependent Na^+^/Ca^2++^ exchanger
7-N: 7-Nitroindazole.
